# Social sciences research in neglected tropical diseases 3: Investment in social science research in neglected diseases of poverty: a case study of Bill and Melinda Gates Foundation

**DOI:** 10.1186/1478-4505-9-2

**Published:** 2011-01-06

**Authors:** Subhash Pokhrel, Daniel Reidpath, Pascale Allotey

**Affiliations:** 1 Health Economics Research Group, Brunel University, UK; 2 Global Public Health, School of Medicine, Monash University, Malaysia

## Abstract

**Background:**

The level of funding provides a good proxy for the level of commitment or prioritisation given to a particular issue. While the need for research relevant to social, economic, cultural and behavioural aspects of neglected tropical diseases (NTD) control has been acknowledged, there is limited data on the level of funding that supports NTD social science research.

**Method:**

A case study was carried out in which the spending of a major independent funder, the Bill and Melinda Gates Foundation (BMGF) - was analysed. A total of 67 projects funded between October 1998 and November 2008 were identified from the BMGF database. With the help of keywords within the titles of 67 grantees, they were categorised as social science or non-social science research based on available definition of social science. A descriptive analysis was conducted.

**Results:**

Of 67 projects analysed, 26 projects (39%) were social science related while 41 projects (61%) were basic science or other translational research including drug development. A total of US$ 697 million was spent to fund the projects, of which 35% ((US$ 241 million) went to social science research. Although the level of funding for social science research has generally been lower than that for non-social science research over 10 year period, social science research attracted more funding in 2004 and 2008.

**Conclusion:**

The evidence presented in this case study indicates that funding on NTD social science research compared to basic and translational research is not as low as it is perceived to be. However, as there is the acute need for improved delivery and utilisation of current NTD drugs/technologies, informed by research from social science approaches, funding priorities need to reflect the need to invest significantly more in NTD social science research.

## Introduction

The neglected tropical diseases (NTDs) are a group of 13 parasitic and bacterial infections (e.g. ascariasis, lymphatic filariasis, leprosy and trachoma). They are major disabling conditions affecting mostly the world's poorest people [1]. NTDs are preventable infectious diseases that had been neglected by major stakeholders including media, governments and organisations working in the health sector. This neglect, coupled with poverty and inadequate treatment and control programmes, aggravated the impact of these diseases in very deprived communities [2]. Not surprisingly, the research in NTDs had also been neglected. A critical reason for this has been the lack of funding to advance research and development for neglected disease control. A recent publication estimates that although just over US$2.5 billion was invested into research and development (R&D) for diseases of poverty, 80% of this funding went into the big three HIV/AIDS, TB and Malaria [3]. Furthermore, most of this funding went into the development of drugs and vaccines. To address the lack of focus on research for NTDs a number of advocates and stakeholders have partnered with national ministries of health and pharmaceutical industries towards the control or elimination the most prevalent NTDs [1]. Similar initiatives have led to a sudden surge in non-commercial R&D in NTDS post 2000 [4] and there are currently about 92 public-private partnerships devoted in this area [5].

The neglected areas of research are interfaces between basic science research, product development research, implementation research, other policy and social science research and, last but not the least, the actual large scale disease control programmes themselves [6]. While research on each of these interfaces is critical, the global research effort on NTDs has been diverse and fragmented with a great imbalance in the areas of coverage [3,7,8].

The scale of social science research in the neglected tropical diseases (NTDs) is particularly fragmented and less prominent compared to other areas of research (see [9,10] in this series). Some argue that there is already a significant extant body of research in the social sciences on NTDs; however there is also a perception that these studies are not on a par with research on other diseases [11,12]. That there is a role for social science research generally is not in doubt - recent discussions on the breakthroughs in R&D for neglected diseases suggest that there are, for instance, opportunities for better-targeted policies extending beyond drug discovery [4]. Areas highlighted include social research on community diagnosis, community participation in the frontline and implementation research [13]. Although these constitute an attempt to articulate a research agenda, there is still no coherent theme or research priority area identified from the social sciences that could lead to a cumulative body of evidence relevant to NTD control. A common reason given for this is the relatively limited funding specifically for social science research in NTDs. Global health trends and the advocacy for NTDs clearly demonstrate that the availability of funding is a powerful driver of research agenda.

Moran et al. provide the most comprehensive data related to neglected disease research and development [3]. According to them, just over US$ 2.5 billion was invested into R&D of new neglected disease products in 2007, a majority (80%) of which, as previously mentioned, went to the three big diseases- HIV/AIDS, TB and malaria. This funding was heavily focussed on drugs and vaccines. Platform technologies that included vaccine adjuvants, diagnostic and delivery technologies received less than 0.4% of the total R&D spending. The major donors were public and philanthropic institutions that invested 69% and 21% of the total spending respectively. About 9% of the funding came from the private pharmaceutical sector. These data provide information about the scale and the nature of NTD research and development. They highlight for instance that research in this area is funded primarily by the BMGF and National Institutes of Health (NIH) in the US. Most of the NIH research dollars are spent on basic science and translational research to bring new drugs and technologies to the market [14]. For conditions prevalent in developing countries such as that related to child mortality, 97% of NIH and Gates monies were in fact spent on creating new technologies [15].

In this paper, we attempt to aid the debate whether both the level and spread of social science research funding is adequate relative to creating new technologies by analysing the expenditure of a leading funder in the area of NTD research and development. This is a part of a larger study looking at the current status of social science research in neglected tropical diseases (see [9,10,16] in this series). While a wider survey including all funders could have been an ideal approach, tracking funding sources specifically for social science research is complex and information provided through the publications is inconsistent [17]. Many authors do not identify their sources of funding in the published articles. Even in instances where funding for the research is acknowledged, stating the amount received is not a usual practice. Therefore, we chose an alternative approach of assessing funding flows in to the social science research in NTDs focusing on one lead funding organisation- the Bill and Melinda Gates Foundation or BMGF (Additional file 1: Box 1). It is the second largest funder of NTD research and development as found by Moran et al. [3] and the data on BMGF funding grantees is openly available. The most obvious limitation with this approach however is that it was sometimes difficult to distinguish between funding allocated specifically to social science research and funds allocated for the implementation of programmes and for the evaluation of those programmes. In addition, even though we focused on BMGF and examined its funding mechanism, we acknowledge that this does not give a comprehensive picture. It does however provide a significant proportion of funding for social science research on NTDs globally. We excluded other funders because it was not feasible to track their funding in the way that allowed us to make any distinction between social science and non-social science research in the NTDs.

## Methods

We searched the BMGF database online. In order to find the relevant social science studies, we first went to the "Search Past Grants" site available at http://www.gatesfoundation.org/grants/Pages/search.aspx which lists all grants that the Foundation has made in the past years. We then filtered the search by checking respectively the "neglected diseases", "2007 and earlier" and "2008" boxes found on the left hand side menu. The search was carried out in November 2009. A total of 68 titles were identified. These included grants between October 1998 and November 2008. The identified grant titles had information about the purpose of the projects, date when it was awarded, amount and term of the grant, the region the grant served, the BMGF programme under which it was allocated (all Global Health) and the location of the Grantee.

Based on this data, we identified keywords within the very limited information contained in the titles and purpose fields of identified grantees and then categorised them as social science research or non-social science research. The basis of the categorisation was the definition of social science provided in a recent report [18] which states,

"[T]he relevance of these [social science] disciplines to public health and disease control is in ... the applied areas of anthropology, demography, economics, human geography, psychology, politics, history, law, social policy, and sociology". [18]

As it was not always possible to identify in the titles the specific discipline under which the identified grant operated, we further utilised a more specific definition given in the same report-

"Applied social science for public health is ...defined as an interdisciplinary and dynamic field which integrates the knowledge and tools for research and analysis from a range of social science disciplines for the purposes of understanding the various determinants of health in individuals and populations and developing, implementing and evaluating sustainable solutions to public health problems".[18]

To illustrate the process of categorisation, consider two examples. Two of the identified titles had the following information on their purpose fields: "to determine the impact of integrated trachoma and lymphatic filariasis control programs on infection prevalence." and "to develop highly effective, inexpensive new drugs to treat late stages of trypanosomiasis and leishmaniasis". Based on the definition of social science research as described above, the former was identified as a social science research as the project was more likely to sit in one or more of the disciplines identified in the definition. The latter was a non-social science research as it was a drug development project.

Other variables of interests included some basic information about the grantees (institutional type, location, amount received, term of award, and year) as well as the beneficiary's region. To compare the funds received for various purposes on relative basis, we generated another indicator- the total grant received per calendar year. A descriptive analysis was carried out by stratifying the total number of projects into two: (a) social science research; and (b) non-social science research.

## Results

For the period 1998 to 2008, a total of 68 projects related to NTD's were funded by the BMGF. After screening the purpose of the funding, 67 projects were identified and the available data on those projects were analysed. One project - a scholarship program aimed at encouraging students to pursue university degrees that are not necessarily NTD-based - was excluded because it was difficult to decipher its direct link to NTD's. Additional file 2 and 3 (Appendices 1 and 2) list the projects selected for this analysis.

**Figure 1:**
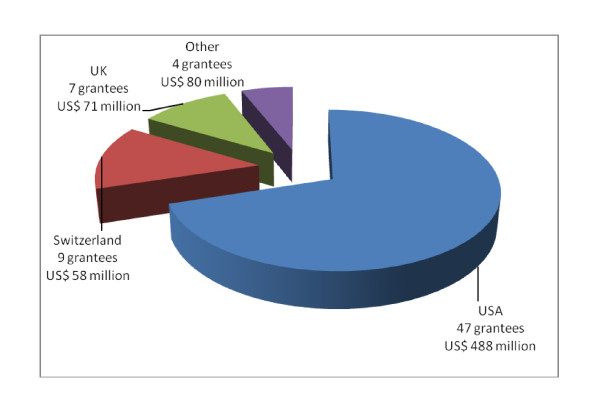
**Location of Grantees**.

The 67 projects were from 5 different continents. Majority (55%) of projects were from Africa followed by Asia (28%) with the remaining covering Europe, as well as North and South America. However, the grantees were mainly located in the US (76%), Switzerland (13%) and the UK (10%). As shown in Figure 1 most (61%) of the projects involved research related to non-social science with the main focus on drug development. About 64% (n = 43) of the projects were associated with academic research institutions while the rest were related to non-government organisations (33%) and other institutions (3%).

**Figure 2:**
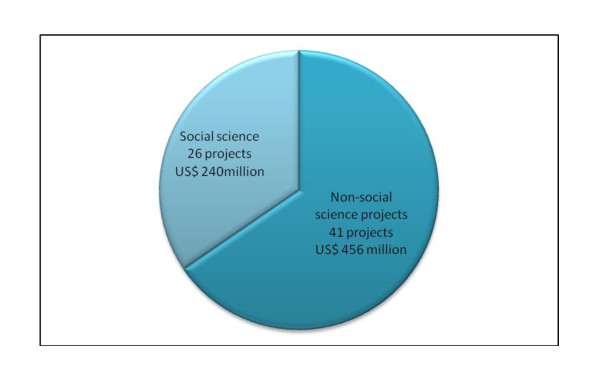
**NTD Social science research funded by Bill & Melinda Gates Foundation 1998-2008**.

During the review period, a total of US$ 697 million was allocated to fund the projects, with amounts ranging from US$ 33,150 to US$ 55 million dispensed for projects that lasted from 4 months to about 10 years. Of these US$ 456 million were allocated to research related to non-social science and US$ 241 million to social science research (Figure 2). In terms of average funds allocated per year, social science research received US$ 1.97 million compared with US$ 2.35 million that went to non-social science research. Although the level of funding for social science research has generally been lower than that for non-social science research over 10 year period, social science research attracted more funding in 2004 (US$ 14.97 million) and 2008 (US$ 45.84 million) compared to non social science research (US$ 0.57 and 8.72 million respectively in 2004 and 2008).

**Figure 3:**
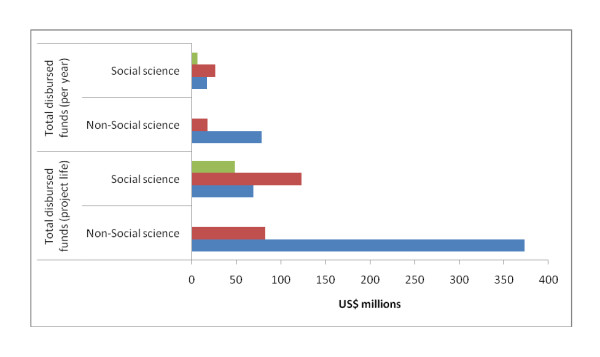
**Bill & Melinda Gates Foundation disbursements by type of grantees (1998-2008)**. Red: Non-governmental Organisation. Blue: Academic and Research Institutes. Green: Others.

In order to review the range of recipients of the funds, the grantees were divided into three broad groups- (a) academic and research institutions (ARI) that included Universities and research centres; (b) non-governmental organisations (NGO) that included not-for-profit institutions; and (c) others that included institutions that were not ARIs or NGOs. As shown in Figure 3 most of the funding for social science research was allocated to non-governmental organisations while academic research institutes were the biggest beneficiaries in terms of receiving funds for non-social science research.

## Discussion

This paper presented a case study of social science funding based on one lead funding organisation- the Bill and Melinda Gates Foundation. The reliance on one funder to provide an indication of the proportion of total research spending going to social science research may not have given us the true picture of the scale of funding in the NTD social science research. However, it does constitute a systematic attempt to explore the commonly held assumption that funding allocated to social science research is limited. Including all other funders in the analysis could have improved the precision of the scale of funding but it might not have altered the observed proportion of such funding and more importantly the conclusion that there exists a noticeable difference between social and non-social science research dollars spent on NTDs. Another limitation was that some of the projects which we considered as social science research may also have programmatic or implementation components. Due to limited information available, it was not possible to separate research costs from programme costs. It does however demonstrate that investment in social science *research *is less than whatever estimates could be gleaned from this case study.

An important finding from this case study is that central to increased discussions on the need for NTD control is the primacy given to basic and translational research into new drugs and technological development. This notwithstanding the evidence that improving the "fidelity" of health care delivery and utilisation (predominantly a social science approach) actually raises the population health status significantly more than the research to develop a more efficacious new drug or technology [14,15]. Given the compelling data on the existing cost effective drugs and technologies to treat NTDs at a unit cost of about US$0.50, so little effort appears to be going into the sorts of questions that will enhance the delivery of these technologies to the target populations. Thus, there is a need for intensified advocacy for more funding to be made available for social science evidence to support this effort. However, this need not imply that cuts in basic/translational research are necessary to do this as it is not clear whether the scale of funding disparities observed in this case study between social-science and basic/translational research is appropriate enough to warrant that.

The pattern of funding from the BMGF provides some interesting implications. Although almost all debates around funding research in NTDs revolve around drug/technology development, the notion that social science research in the NTDs receives significantly less funding relative to research in to basic science and drug development may be exaggerated, particularly over the last five to ten years. In the case of BMGF, this may have been due to the fact that the BMGF aims to support sustainable ways to improve delivery where proven tools exist [2]. This goal is also reflected in the partnerships and NGOs to realise the benefits of existing tools. However, the gap in social versus non-social science research funding is still significant (35/65). Given the acute need for improved delivery and utilisation of current drugs/technologies, informed by research from social science approaches, funding priorities need to reflect the need to invest significantly more in social science. At the very least there is an urgent need for an assessment and debate around the opportunity costs of investing any future dollars in developing a new NTD drug or technology.

We observed a couple of interesting disparities in funding social science research in the case of BMGF. First, academic/research environment received less funding compared to NGOs. This could be explained by the fact that most of such research actually required the delivery/implementation of current tools (drugs/therapy/technology). NGOs and partnerships, many of whom are involved in direct service delivery, are better placed to undertake these activities. However, this observation should be located in an important caveat of our method of this case study- our inability to make a clear distinction in some cases between funding allocated specifically to social science research and funds allocated for the implementation of programmes and for the evaluation of those programmes. Some NGOs might have just been implementing current tools as opposed to doing research. Nevertheless, future funding decisions may consider whether a partnership between social science researchers from the academia/research institutions and the NGOs may provide a better model (see [10,16] in this series). Secondly, funds for projects in lower and middle income countries are mainly awarded to institutions based in high-income countries. This raises yet another important but traditionally identified issue- the lack of research capacity in low-income countries, the home of NTDs. We were unable to explore this issue further in this case study.

Finally, the level of funding provides a good proxy for the level of commitment or prioritisation given to a particular issue [19]. It is therefore difficult to argue that the social, economic, cultural and behavioural aspects of NTD control are considered important to address when the funding is not made available to support it. The other analyses presented in this series of papers [9,10] show that the nature of projects that are funded focus on support for existing interventions through evaluation, understanding community drivers, health systems bottlenecks, and in broad programmatic areas although there is still extensive, systematic work that needs to be undertaken in these areas. Furthermore, these analyses also show that the full potential of the possible contribution that the wide range of social science disciplines could make to neglected populations has been barely tapped [9]. This means that a clear social science agenda and some good advocacy for social science research on NTDs are needed to attract more research funding to this area.

## Conclusion

The evidence presented in this case study indicates that the notion that social science research in the NTDs receives significantly less funding relative to research in to basic science and drug development may be exaggerated, particularly over the last five to ten years. However, as there is the acute need for improved delivery and utilisation of current drugs/technologies, informed by research from social science approaches, funding priorities need to reflect the need to invest significantly more in social science research. Future research could look at how best this goal can be achieved- through cuts in any future dollars in developing a new NTD drug/technology or increased investment in social science studies independent of basic/translational research.

## Competing interests

The authors declare that they have no competing interests.

## Authors' contributions

SP carried out the data analysis and constructed first draft of the manuscript with subsequent inputs and revisions from DR and PA. All authors read and approved the final manuscript.

## Supplementary Material

Additional file 1 **Box 1: The Bill & Melinda Gates Foundation**. This box provides a short introduction to Bill & Melinda Gates Foundation and briefly describes what role the Foundation plays on funding global health research. Click here for file

Additional file 2 **Appendix 1: Bill & Melinda Gates Foundation disbursements for social science research 1998-2000 (extracted from Gates Foundation database)**. This appendix provides a list of research projects funded by the Bill & Melinda Gates Foundation between 1998 and 2000. These projects have been classified as social science research in this paper. Click here for file

Additional file 3 **Appendix 2: Bill & Melinda Gates Foundation disbursements for non-social science research 1998-2000 (extracted from Gates Foundation database)**. This appendix provides a list of research projects funded by the Bill & Melinda Gates Foundation between 1998 and 2000. These projects have been classified as non-social science research in this paper. Click here for file
