# Supplementary material for: Social sciences research in neglected tropical diseases 3: Investment in social science research in neglected diseases of poverty: a case study of Bill and Melinda Gates Foundation
Source: Health Res Policy Syst. 2011 Jan 6;9:2. doi: 10.1186/1478-4505-9-2 (PMC3022559; doi:10.1186/1478-4505-9-2)
Supplement: Additional file 1 — Box 1: The Bill & Melinda Gates Foundation. This box provides a short introduction to Bill & Melinda Gates Foundation and briefly describes what role the Foundation plays on funding global health research. [file 1478-4505-9-2-S1.DOC]

**Box 1: The Bill & Melinda Gates Foundation**

The BMGF has invested in exploring methods that are effective and affordable for the treatment or elimination of severe impacts that NTDs have, particularly on the people living in low-income countries. It provides funding to various public, private and non-profit organizations. Under its Global Health Programme, BMGF “advances in science and technology to save lives in poor countries”. According to BMGF, the focus has been on the “health problems that have a major impact in developing countries but get too little attention and funding”. There are two ways of support – First, supporting “sustainable ways to improve delivery where proven tools exist and where they don’t, investing in research and development of new interventions, such as vaccines, drugs, and diagnostics” (2). The BMGF funds a very significant portion of global health research on neglected diseases, amounting to USD 452 million which is 17.7% of total global funding (3).
