# Supplementary material for: Social sciences research in neglected tropical diseases 3: Investment in social science research in neglected diseases of poverty: a case study of Bill and Melinda Gates Foundation
Source: Health Res Policy Syst. 2011 Jan 6;9:2. doi: 10.1186/1478-4505-9-2 (PMC3022559; doi:10.1186/1478-4505-9-2)
Supplement: Additional file 2 — Appendix 1: Bill & Melinda Gates Foundation disbursements for social science research 1998-2000 (extracted from Gates Foundation database). This appendix provides a list of research projects funded by the Bill & Melinda Gates Foundation between 1998 and 2000. These projects have been classified as social science research in this paper. [file 1478-4505-9-2-S2.DOC]

Appendix 1: Bill & Melinda Gates Foundation disbursements for social science research 1998-2000 (extracted from Gates Foundation database)

| **Grantee** | **Year** | **US$, million** | **Region** | **Purpose of the grant** | **Website** |
| --- | --- | --- | --- | --- | --- |
| The Carter Centre | 2008 | 40.00 | Africa | for Global Campaign to Eradicate Guinea Worm Disease | http://www.cartercenter.org |
| IDSA Education and Research Foundation | 2008 | 1.96 | North America | to promote a robust US response for combating HIV/AIDS and tuberculosis by establishing an Infectious Disease | n/a |
| Albert B. Sabin Vaccine Institute, Inc. | 2008 | 3.88 | Africa, Asia, South America | to promote advocacy and resource mobilization for the seven most prevalent NTDs: ascariasis, hookworm infecton. | http://www.sabin.org |
| Imperial College London | 2007 | 0.05 | Africa | to fund a workshop to identify the impact of genital schistosomiasis (male and female) on public health and.. | http://www.imperial.ac.uk |
| Johns Hopkins University | 2007 | 12.04 | Africa | to address recurrence of trichiasis following surgery and the likely trajectory of elimination of blinding | http://www.jhsph.edu |
| The Henry M. Jackson Foundation for Advancement of Military Medicine | 2007 | 3.53 | Asia, South America | to develop an effective vector behaviour-modifying strategy for the prevention of dengue in Peru and Thailand | http://www.hjf.org |
| University of California San Francisco | 2007 | 5.00 | Global | for general operating support for the Global Health Group | n/a |
| The Task Force for Child Survival and Development | 2006 | 11.71 | Global | to resolve the critical challenges now facing the global program to eliminate lymphatic filariasis | n/a |
| International Trachoma Initiative | 2006 | 10.11 | Africa | to determine the impact of integrated trachoma and lymphatic filariasis control programs on infection prevalence. | http://www.trachoma.org |
| The Carter Centre | 2006 | 5.26 | Africa, Global | to integrate malaria and lymphatic filariasis (LF) programs in seven South-Eastern states of Nigeria to demo.. | http://www.cartercenter.org |
| Imperial College London | 2006 | 9.98 | Africa | to support development, implementation and evaluation of an integrated health package for poor populations.. | http://www.imperial.ac.uk |
| World Health Organization | 2006 | 5.03 | Global | to coordinate the development and adoption of international public health policies aimed at integrating dis.. | http://www.who.int |
| Public Library of Science | 2006 | 1.09 | Global | to create a sustainable and open access journal that addresses neglected tropical diseases | n/a |
| Program for Appropriate Technology in Health (PATH) | 2005 | 5.00 | North America, Global | for general operating support | http://www.path.org |
| World Health Organization | 2005 | 2.41 | Africa, Global | to develop integrated community-directed interventions against diseases of neglected populations in Africa | http://www.who.int |
| Program for Appropriate Technology in Health (PATH) | 2005 | 0.33 | Global | to increase global support for HPV, Japanese encephalitis and haemophilus influenza type B in developing c.. | http://www.path.org |
| The Carter Centre | 2005 | 25.00 | Africa, Global | to support the eradication of Guinea Worm Disease in the remaining endemic countries | http://www.cartercenter.org |
| Nuclear Threat Initiative | 2004 | 2.25 | Global | to support a disease outbreak detection program | http://www.nti.org |
| World Health Organization | 2004 | 2.06 | Africa, Global | to assess the feasibility of onchocerciasis elimination by ivermectin treatment in Africa | http://www.who.int |
| The Carter Centre | 2004 | 10.66 | North America, South America, Global | to enable the eradication and certification of onchocerciasis eradication in the Americas and extending this. | http://www.cartercenter.org |
| World Health Organization | 2002 | 2.00 | Global | to fund WHO support for building global partnerships for schistosomiasis and intestinal worm | http://www.who.int |
| Emory University | 2002 | 0.33 | Africa, Asia, North America, South America, Global | to support a U.S. committee to increase support for lymphatic filariasis elimination | n/a |
| Imperial College London | 2002 | 31.93 | Africa, Global | to support the Schistosomiasis Control Initiative (SCI) in Africa | http://www.imperial.ac.uk |
| International Bank for Reconstruction and Development | 2000 | 20.00 | Africa, Asia, Global | to support the Global Alliance for the Elimination of Lymphatic Filariasis | http://www.worldbank.org |
| President and Fellows of Harvard College | 2000 | 0.73 | Africa, Global | to support the Schistosomiasis Control Initiative | http://www.harvard.edu |
| International Bank for Reconstruction and Development | 2000 | 28.49 | Africa, Global | to support the Dracunculiasis Eradication Trust Fund, in order to facilitate the remaining activities required. | http://www.worldbank.org |

Source: http://www.gatesfoundation.org/grants/Pages/search.aspx
