# Supplementary material for: Social sciences research in neglected tropical diseases 3: Investment in social science research in neglected diseases of poverty: a case study of Bill and Melinda Gates Foundation
Source: Health Res Policy Syst. 2011 Jan 6;9:2. doi: 10.1186/1478-4505-9-2 (PMC3022559; doi:10.1186/1478-4505-9-2)
Supplement: Additional file 3 — Appendix 2: Bill & Melinda Gates Foundation disbursements for non-social science research 1998-2000 (extracted from Gates Foundation database). This appendix provides a list of research projects funded by the Bill & Melinda Gates Foundation between 1998 and 2000. These projects have been classified as non-social science research in this paper. [file 1478-4505-9-2-S3.DOC]

**Appendix 2: Bill & Melinda Gates Foundation disbursements for non-**social science research 1998-2000 (extracted from Gates Foundation database)

| **Grantee** | **Year** | **US$, million** | **Region** | **Purpose of the grant** | **Website** |
| --- | --- | --- | --- | --- | --- |
| Fraunhofer USA, Inc. | 2005 | 1.46 | Africa, Global | to support a pre-clinical assessment for the development a cost effective African Trypanosomiasis vaccine | n/a |
| University of North Carolina at Chapel Hill | 2006 | 21.27 | Africa, Asia, South America, Global | to develop highly effective, inexpensive new drugs to treat late stages of trypanosomiasis and leishmaniasis | http://www.unc.edu |
| Albert B. Sabin Vaccine Institute, Inc. | 2000 | 18.00 | Asia, South America, Global | to develop a genetically engineered recombinant hookworm vaccine | http://www.sabin.org |
| Program for Appropriate Technology in Health (PATH) | 2003 | 35.74 | Asia, Global | to support accelerating vaccine access for Japanese encephalitis | http://www.path.org |
| Helen Keller International | 1999 | 5.00 | Africa, Asia, Global | to expand trachoma elimination and support programs in China, Burkina Faso, Cambodia, Mali, Morocco, Nepal, Niger, Tanzania, and Viet Nam, increasing their sustainability in addressing the significant burden of trachoma and blindness | n/a |
| FIND | 2007 | 3.92 | Africa | to support mapping the distribution of Human African Trypanosomiasis | http://www.finddiagnostics.org |
| Albert B. Sabin Vaccine Institute, Inc. | 2006 | 13.79 | South America, Global | to produce an effective, manufacturable, affordable and sustainable Human Hookworm Vaccine (HHV) | http://www.sabin.org |
| Institute for One World Health | 2005 | 30.00 | Asia, Global | to help avert visceral leishmaniasis-related morbidity and mortality in South Asia | http://www.oneworldhealth.org |
| Institute for One World Health | 2002 | 7.25 | Asia, Global | to support the drug development program for visceral leishmaniasis | http://www.oneworldhealth.org |
| International Society for Infectious Diseases | 2007 | 0.25 | Africa, Asia | to support the professional development of women professionals from low-income countries in the field of infectious diseases by enabling participation at the 13th International Congress on Infectious Diseases | http://www.isid.org |
| Albert B. Sabin Vaccine Institute, Inc. | 2005 | 21.87 | Africa, Asia, South America, Global | to support the Human Hookworm Vaccine Initiative | http://www.sabin.org |
| Liverpool School of Tropical Medicine | 2006 | 4.20 | Africa | to prevent disease (Human African Trypanosomiasis) through research to develop an effective vector control strategy | http://www.liv.ac.uk/lstm/ |
| The Carter Centre | 2006 | 5.58 | Africa | to develop a model for integrated health interventions and demonstrate in Nigeria that the model is feasible, replicable, and effective at controlling multiple priority diseases | http://www.cartercenter.org |
| Albert B. Sabin Vaccine Institute, Inc. | 2003 | 0.08 | Global | to support a colloquia on the topic of chlamydia vaccines | http://www.sabin.org |
| Swiss Tropical Institute | 2006 | 5.00 | Africa, Asia, Europe, Global | to support STI research and resources platforms for the development, validation and application of public health tools in developing countries | n/a |
| Institute for One World Health | 2005 | 11.69 | Asia, Global | to support the Phase III drug development program for visceral leishmaniasis | http://www.oneworldhealth.org |
| Swiss Tropical Institute | 2007 | 2.00 | Africa | to establish a centre for clinical research in Kinshasa, Democratic Republic of Congo for assessment of new drugs for African Sleeping Sickness | n/a |
| International Medical Corps | 1999 | 0.72 | Africa, Global | to support sleeping sickness surveillance and control in Tambura County, Sudan | http://www.imcworldwide.org |
| Institute for One World Health | 2002 | 0.95 | South America, Global | to support the drug development program for visceral leishmaniasis | http://www.oneworldhealth.org |
| University of North Carolina at Chapel Hill | 2000 | 17.83 | Africa, Global | to support the development of novel drug candidates for the treatment of human African trypanosomiasis and leishmaniasis | http://www.unc.edu |
| Seattle Biomedical Research Institute | 2004 | 0.08 | Africa, Asia, South America, Global | to support the Trypanosomatid Pathogens Genome Meeting | http://www.sbri.org |
| Universidad Peruana Cayetano Heredia | 2006 | 0.60 | South America, Global | to provide supplemental training funds for a demonstration project to eliminate cysticercosis | http://www.upch.edu.pe |
| The Henry M. Jackson Foundation for the Advancement of Military Medicine | 2003 | 1.06 | Global | to support a development plan for a Molecular Tetravalent Dengue Vaccine for the Global Prevention of Dengue Fever, Dengue Hemorrhagic Fever, and Dengue Shock Syndrome | http://www.hjf.org |
| University of Notre Dame | 1999 | 5.21 | North America, Global | to eliminate lymphatic filariasis in Haiti while developing a model program for the global elimination of this infection | http://www.nd.edu |
| International Vaccine Institute | 2003 | 55.00 | Asia, Global | to fund effective and affordable dengue vaccines for children in dengue-endemic areas | http://www.ivi.org |
| University of Kentucky Research Foundation | 2007 | 5.38 | Global | to eliminate Lymphatic Filariasis (LF) in the South Pacific, using anti-vector approaches that will directly impact dengue transmission and are transferable to multiple mosquito-borne disease | n/a |
| International Trachoma Initiative | 1998 | 1.00 | Africa, Asia | to eliminate trachoma-related blindness in Morocco, Tanzania, Ghana, Mali, and Viet Nam | http://www.trachoma.org |
| Liverpool School of Tropical Medicine | 2007 | 23.74 | Global | to fund the establishment of anti-symbiotic chemotherapy directed against Wolbachia bacterial endosymbionts for the control and treatment of human filariasis | http://www.liv.ac.uk/lstm/ |
| Makerere University | 2007 | 0.04 | Africa | to convene a group of experts who will consider the role of xenomonitoring and the characteristics of a tool that would be developed for such a use | n/a |
| Drugs for Neglected Diseases initiative | 2007 | 25.73 | Africa | to develop two new drug candidates for HAT (one lead and one back-up) through the preclinical stage to where they are ready to enter phase I human clinical trials; and to select one drug candidate for phase I, normal volunteer, clinical studies | n/a |
| Universidad Peruana Cayetano Heredia | 2003 | 15.53 | South America, Global | to eliminate cysticercosis and taeniasis from an endemic area in Peru | http://www.upch.edu.pe |
| University of North Carolina at Chapel Hill | 2006 | 18.58 | Africa, Global | to conduct Phase III trials and obtain regulatory approval for an oral drug for early stage African sleeping sickness | http://www.unc.edu |
| Infectious Disease Research Institute | 2006 | 32.06 | Global | to eliminate leishmaniasis by developing a vaccine for use in both treatment and prevention | http://www.idri.org |
| Fraunhofer USA, Inc. | 2007 | 6.96 | Africa | to support the transfer of technology and potential products on reasonable terms regarding African trypanosomiasis | n/a |
| University of Notre Dame | 2006 | 4.43 | North America | to provide essential treatment for lymphatic filariasis (LF) and intestinal helminths with the goal of eliminating LF from Haiti | http://www.nd.edu |
| Infectious Disease Research Institute | 2000 | 15.00 | Global | to develop a vaccine for leishmaniasis by focusing on the scientific and practical issues leading to the development of effective vaccines and therapies, initially in Brazil and India | http://www.idri.org |
| FIND | 2005 | 9.86 | Africa, Global | to develop and evaluate diagnostic tests to support the control of Human African Trypanosomiasis in Africa | http://www.finddiagnostics.org |
| Makerere University | 2008 | 8.69 | Africa | to develop and apply xenomonitoring tools in human African Trypanosomiasis control programs in developing countries | n/a |
| Keystone Symposia on Molecular and Cellular Biology | 2004 | 0.49 | Global | to connect the scientific community with conferences on global infectious diseases to catalyze information exchange and translation of basic science into new therapies | http://www.keystonesymposia.org |
| Gordon Research Conferences | 2008 | 0.03 | Africa, Asia, South America | for conference support | http://www.grc.org |
| The Task Force for Global Health, Inc. | 2000 | 20.00 | Africa, Asia, Global | to eliminate blinding trachoma in 10 countries and to further improve the tools for fighting trachoma | http://www.taskforce.org |

Source: http://www.gatesfoundation.org/grants/Pages/search.aspx
